# Short-term outcomes of mirogabalin in patients with peripheral neuropathic pain: a retrospective study

**DOI:** 10.1186/s13018-020-01709-3

**Published:** 2020-05-26

**Authors:** Tomoko Tetsunaga, Tomonori Tetsunaga, Keiichiro Nishida, Haruo Misawa, Tomoyuki Takigawa, Kentaro Yamane, Hironori Tsuji, Yoshitaka Takei, Toshifumi Ozaki

**Affiliations:** 1 Department of Orthopedic Surgery, Okayama University Hospital, Okayama, Japan, 2-5-1 Shikata-cho, Kitaku, Okayama City, Okayama, 700-8558 Japan; 2 Department of Orthopedic Surgery, Kurashiki Municipal Hospital, 2-39, Kojima-Ekimae, Kurashiki, 711-0921 Japan

**Keywords:** Peripheral neuropathic pain, Mirogabalin, Pregabalin, Adverse event

## Abstract

**Background:**

Mirogabalin, which is approved for the treatment of peripheral neuropathic pain in Japan, is a ligand for the α2δ subunit of voltage-gated calcium channels. Both pregabalin and mirogabalin act as nonselective ligands at the α2δ-1 and α2δ-2 subunits. Mirogabalin has a unique binding profile and long duration of action. Pregabalin has been reported to produce intolerable adverse effects in some patients. This study investigated outcomes associated with mirogabalin administration in patients with peripheral neuropathic pain who ceased treatment with pregabalin.

**Methods:**

We retrospectively assessed peripheral neuropathic pain using the neuropathic pain screening questionnaire (NeP score) in 187 patients (58 men, 129 women) who were treated with mirogabalin. All patients had switched from pregabalin to mirogabalin due to lack of efficacy or adverse events. Differences in the treatment course (i.e., numeric rating scale (NRS) scores) were compared using one-way analysis of variance with Bonferroni post hoc tests.

**Results:**

The mean age of the patients was 72.3 years (range, 30–94 years), and the mean duration of disease was 37 months (range, 3–252 months). After treatment with mirogabalin for 1 week, NRS scores significantly decreased compared with baseline and continued to decrease over time. After 8 weeks, NRS scores improved by ≥ 30% from baseline in 113 patients (69.3%). Twenty-four patients (12.8%) stopped mirogabalin treatment due to adverse events. Somnolence (26.7%), dizziness (12.3%), edema (5.9%), and weight gain (0.5%) were noted as adverse events of mirogabalin.

**Conclusions:**

The results of this investigation indicate that mirogabalin is safe and effective for reducing peripheral neuropathic pain.

## Background

The International Association for the Study of Pain defined neuropathic pain as “pain caused by a lesion or disease of the somatosensory nervous system” [1]. Neuropathic pain results in multiple symptoms, including spontaneous neurological pain, allodynia, hyperalgesia, and numbness, and results in decreases in quality of life (QOL) [2]. In addition, neuropathic pain may become intractable [2]. Chronic low back pain is known to be due to neuropathic as well as nociceptive pain mechanisms [3, 4]. Patients with neuropathic pain show higher ratings for pain intensity with more comorbidities, such as depression panic/anxiety disorder, and sleep disorders than those with nociceptive pain [4]. It is thus important to determine which factors contribute to neuropathic pain at an early stage and start appropriate drug therapy [5]. Unfortunately, current treatment methods are not always satisfactory [5].

Pregabalin is a ligand for the α2δ subunit of voltage-sensitive calcium channels and is recommended as the first-line drug for neuropathic pain in guidelines around the world [6]. It decreases the release of neurotransmitters such as glutamate, noradrenalin, and substance P, which leads to pain relief [7]. Pregabalin has been used in patients with neuropathic pain and shown to be a cost-effective treatment [8, 9] that has a positive impact on QOL [10, 11]. It is generally well tolerated [12], and most adverse events are mild to moderate [13]. Pregabalin binds to the α2δ-1 and α2δ-2 subunits of presynaptic, voltage-dependent calcium channels, which are widely distributed throughout the central and peripheral nervous systems [14]. The most frequently reported side effects of pregabalin include dizziness and somnolence, which are related to the central nervous system (CNS) [14]. Therefore, the clinical utility of pregabalin may be limited by CNS adverse events [14].

Mirogabalin, which is a potent and specific ligand for the α2δ subunit of voltage-gated calcium channels, is an orally administered gabapentinoid developed for the treatment of peripheral neuropathic pain in Japan [15, 16]. This agent has the distinguishing feature of persistently binding to the α2δ-1 subunit, which plays an important role in neuropathic pain [16]. This new drug is reportedly well tolerated and well absorbed following oral administration and was first approved for peripheral neuropathic pain in 2019.

Although there are many positive reports associated with pregabalin, some patients have moderate adverse events [14]. Tetsunaga et al. reported that somnolence (46.9%), dizziness (18.8%), weight gain (9.4%), and rash (4.7%) were noted as side effects of pregabalin [17]. No reports regarding the treatment outcomes with mirogabalin in patients who experienced moderate adverse events with pregabalin treatment have been published. In the present study, we examined the outcomes with mirogabalin as a rescue drug in patients with peripheral neuropathic pain who developed moderate side effects from pregabalin treatment.

## Methods

### Participants

This retrospective study included outpatients with peripheral neuropathic pain who consulted our hospital between April 2019 and October 2019. Data were collected from medical records. The diagnosis of peripheral neuropathic pain was based on a history of neuropathic pain and confirmatory findings on examination. The inclusion criteria were a diagnosis of peripheral neuropathic pain based on the flow chart of the grading system for neuropathic pain [18], lack of efficacy with pregabalin (Lyrica®, Pfizer Inc., Tokyo, Japan) or adverse events with pregabalin treatment, and the willingness to answer a questionnaire. The exclusion criteria included dementia, delirium, or other conditions that made it difficult to complete a self-reported written questionnaire. Patients with severe chronic diseases that interfered with treatment (e.g., cardiovascular disease, renal failure, or other disqualifying conditions) were also excluded. At baseline, the patients completed a self-reported questionnaire and provided demographic and clinical information. This study was approved by the Kurashiki Municipal Hospital ethics committee, and written informed consent was waived because of the retrospective design.

### Procedure

#### Treatment protocol

At least 1 month after cessation from pregabalin, treatment with mirogabalin (Tarlige®, Daiichi Sankyo, Inc., Tokyo, Japan) was prescribed. The patients received mirogabalin 10 mg/day orally for the first week. In patients with decreased renal function, the dose of mirogabalin was decreased to 5 mg/day. Depending on patient age and symptoms, the dose of mirogabalin was decreased or increased as required to between 2.5 mg and 15 mg/dose twice daily. All patients visited the hospital at 1, 2, 4, and 8 weeks to ensure compliance with the study regimen. The patients with an adequate effect received the same dose of mirogabalin. In patients with an inadequate effect, the dose of mirogabalin was increased up to 30 mg/day. If adverse events were observed, the dose was decreased. In this study, no other conservative treatments (nonsteroidal anti-inflammatory drugs and rehabilitation) or surgeries were performed. During the study, only the dispensing pharmacist had knowledge of the patient codes. The manufacturer and provider of mirogabalin (Daiichi Sankyo, Inc., Tokyo, Japan) was not involved in the protocol development, data collection and management, statistical analysis, or manuscript preparation.

#### Clinical assessment

The neuropathic pain screening questionnaire (NeP score), developed by Ogawa et al., was used for the peripheral neuropathic pain survey (Table 1) [19–21]. The patients’ answers to questions in seven domains were weighted and scored. The likelihood of neuropathic pain was determined based on the total score as follows: ≥ 5 = highly likely to have neuropathic pain; 4 = likely to have neuropathic pain; 3 = possibility of neuropathic pain; ≤ 2 = unlikely to have neuropathic pain. A score ≥ 4 was judged as representing neuropathic pain [20]. The numeric rating scale (NRS) for pain self-assessment is a widely used, valid, and reliable tool to measure chronic pain intensity [22]. The scores ranged from 0 to 10, with 0 representing no pain and 10 representing the worst pain imaginable. The NRS scores were obtained at baseline and at 1, 2, 4, and 8 weeks of treatment. We also evaluated adverse events of mirogabalin.

**Table 1:** Neuropathic pain screening questionnaire

Question	
Q1	There is a pinprick-like pain
Q2	There is an electric shock-like pain
Q3	There is a tingling burning pain
Q4	There is pain with strong numbness
Q5	A light touch with clothing or cold wind causes pain
Q6	The site of pain has decreased or increased sensation
Q7	The site of pain shows skin swelling and/or discoloration to red or purple

Each of the items is scored on a 5-point scale (0 = never; 1 = slight; 2 = moderate; 3 = strong; 4 = very strong). The total score can range from 0 to 28 points, with higher scores indicating greater pain.

#### Primary endpoint

The primary endpoint of this study was to analyze the treatment course of peripheral neuropathic pain using NRS scores in patients receiving mirogabalin.

#### Secondary endpoint

The secondary endpoint of the current study was to investigate the adverse events of mirogabalin.

### Statistical analysis

Factors associated with the cessation of mirogabalin treatment due to adverse events were identified using univariate analyses between the patients who continued treatment and those who ceased from treatment. Differences in the treatment course (i.e., NRS scores) were compared using one-way analysis of variance with Bonferroni post hoc tests. Moderate improvements in pain are considered to be 30%h Bonferroni postnivariate 50% pain relief is considered a good outcome [23, 24]. Therefore, we divided patients into two groups based on pain relief levels of < 30% or ≥ 30% after 8 weeks of treatment with mirogabalin. Normally distributed variables were compared using Student’s t-tests, and non-normally distributed variables were compared using Mann–Whitney U tests. ables were compared using d using Studentpain relief levels of <analyses between ain using NRS scores in pabetween groups. Differences in the magnitude of NRS improvement between the groups with initial doses of 5 mg/day and 10 mg/day were compared using Student’s t-tests. Differences in the magnitude of NRS improvement in patients taking maximum doses were compared using one-way analysis of variance. Differences of *p* < 0.05 were considered significant. Statistical analyses were conducted using SPSS software version 25.0 for Windows (IBM Corporation, Armonk, NY, USA).

## Results

### Participants

Of the 337 outpatients with peripheral neuropathic pain who had been treated with pregabalin, 187 (55.5%) met the inclusion criteria and were included in this study (Table 2). Adverse events leading to the switch from pregabalin to mirogabalin included somnolence (97 patients, 28.8%), dizziness (50 patients, 14.8%), edema (7 patients, 2.1%), weight gain (3 patients, 0.9%), epigastralgia (2 patients, 0.6%), and fatigue (2 patients, 0.6%). Thirty-two patients switched to mirogabalin due to a lack of efficacy with pregabalin. This study included 58 men and 129 women with a mean age of 72.3 years (range, 30–94 years) at the time of the baseline examination. The mean pain duration from onset until consultation was 37 months (range, 3–252 months). In this study, 134 patients had lumbar canal stenosis, 33 had cervical spondylotic myelopathy, 10 had lumbar disc herniation, 9 had carpal tunnel syndrome, and 1 had postoperative pain.

**Table 2:** Patient characteristics

Variables	***n*** = 187
Age (years)	72.3 ± 12.4 (30–94)
Sex, female/male	129/58
Diagnosis	
LCS	134
CSM	33
LDH	10
CTS	9
Others	1
BMI (kg/m^2^)	23.1 ± 4.2 (18–33)
DM	17

Data are expressed as the mean ± standard deviation (range) or *n* (%)

*LCS* lumbar canal stenosis, *CSM* cervical spondylotic myelopathy, *LDH* lumbar disc herniation, *CTS* carpal tunnel syndrome, *BMI* body mass index, *DM* diabetes mellitus

### Treatment with mirogabalin

Fifty-nine patients received mirogabalin 10 mg/day orally for the first week, and 128 patients received mirogabalin 5 mg/day due to decreased renal function. Thirty-three patients were treated with 5 mg/day for 8 weeks of treatment. Eighty-one patients increased the dose of mirogabalin to 10 mg/day due to lack of efficacy. Thirty patients increased the dose of mirogabalin to 20 mg/day. Nineteen patients increased the dose of mirogabalin to 30 mg/day; none of these patients experienced severe adverse events with the increased dose. Twenty-four patients (12.8%) withdrew from mirogabalin treatment because of adverse events. Adverse events associated with mirogabalin included somnolence (50 patients, 26.7%), dizziness (23 patients, 12.3%), edema (11 patients, 5.9%), epigastric pain (2 patients, 1.1%), weight gain (1 patient, 0.5%), and fatigue (1 patient, 0.5%). We investigated factors associated with the cessation of mirogabalin treatment due to adverse events. There were no significant differences in age, sex, body mass index (BMI), or diagnosis between the patients who discontinued mirogabalin and those who continued its use (Table 3). The patients who discontinued mirogabalin had a significantly lower incidence of somnolence (*p* = 0.0017) and significantly higher incidences of dizziness (*p* = 0.015) and edema (*p* = 0.012) with pregabalin than those who continued mirogabalin. The patients who discontinued mirogabalin also had significantly higher incidences of dizziness (*p* = 0.0069) and edema (*p* = 0.016) than the patients who continued mirogabalin, and these effects contributed to the cessation of treatment.

**Table 3:** Univariate analyses comparing factors associated with continuation or withdrawal of treatment with mirogabalin

**Variables**	**Continued treatment** **(*n*= 163)**	**Withdrew from treatment** **(*n*= 24)**	***p*value**
Age (years)	71.8 ± 12.7 (30–94)	75.0 ± 9.9 (49–91)	0.23^a^
Sex, female/male	110/53	19/5	0.25^b^
Diagnosis			0.98^b^
LCS	118	17	
CSM	28	5	
LDH	9	1	
CTS	8	1	
Others	1	0	
BMI (kg/m^2^)	22.9 ± 4.1 (18–33)	23.2 ± 3.9 (19–31)	0.78^a^
DM	15	2	0.89^b^
Adverse events with pregabalin			
Somnolence	92 (56.4%)	5 (20.8%)	0.0017^b^
Dizziness	39 (23.9%)	11 (45.8%)	0.015^b^
Edema	4 (2.5%)	3 (12.5%)	0.012^b^
Weight gain	3 (1.8%)	0 (0%)	0.51^b^
Others	2 (1.2%)	2 (8.3%)	
Lack of efficacy with pregabalin	27 (16.6%)	5 (20.8%)	0.61^b^
NeP score (points)	7.0 ± 1.7 (6–12)	6.9 ± 1.7 (6–12)	0.80^b^
Primary dose of mirogabalin (mg)	3.1 ± 1.2 (5–10)	3.3 ± 1.8 (5–10)	0.52^b^
Adverse events with mirogabalin			
Somnolence	44 (27%)	6 (25%)	0.84^b^
Dizziness	16 (9.8%)	7 (29.2%)	0.0069^b^
Edema	7 (4.3%)	4 (16.7%)	0.016^b^
Epigastric pain	0 (0%)	2 (8.3%)	< 0.0001^b^
Weight gain	1 (0.6%)	0 (0%)	0.70^b^
Fatigue	1 (0.6%)	0 (0%)	0.70^b^

Data are expressed as the mean ± standard deviation (range) or *n* (%).

*LCS* lumbar canal stenosis, *CSM* cervical spondylotic myelopathy, *LDH* lumbar disc herniation, *CTS* carpal tunnel syndrome, *BMI* body mass index, *DM* diabetes mellitus, *NeP* neuropathic pain

^a^ Student’s t-test

^b^ Chi-squared test

**Fig. 1:**
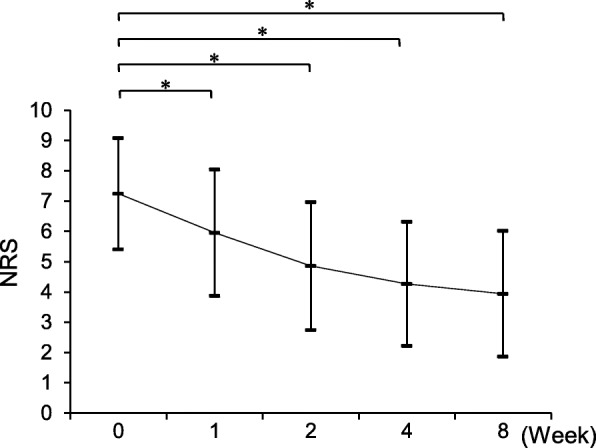
Scores on the numeric rating scale (NRS) were significantly reduced after 8 weeks of treatment with mirogabalin. Data are expressed as the mean ± standard deviation. *p < 0.05

**Fig. 2:**
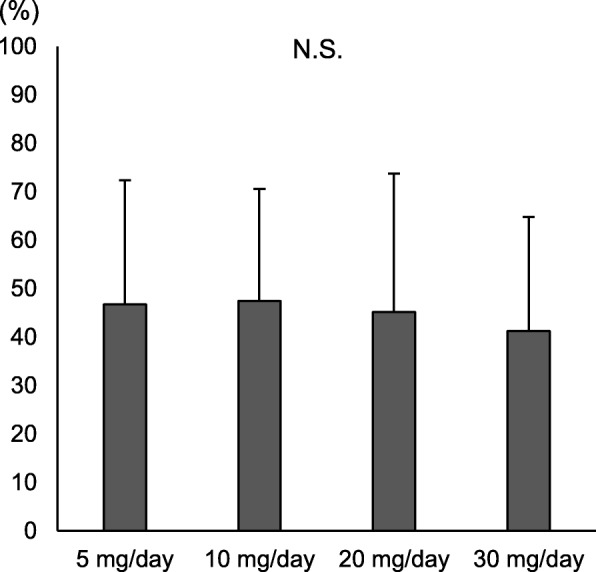
The magnitude of numeric rating scale (NRS) improvement after 8 weeks of treatment with mirogabalin. No significant differences were observed in the magnitude of NRS improvement in patients taking maximum doses of 5 mg/day (*n* = 47), 10 mg/day (*n* = 89), 20 mg/day (*n* = 32), and 30 mg/day (*n* = 19). Data are expressed as the mean ± standard deviation. N.S., not significant

We also investigated changes in NRS scores over the course of treatment with mirogabalin for 8 weeks. After treatment with mirogabalin for 1 week, NRS scores significantly decreased compared with baseline (*p* < 0.0001, Fig. 1) and subsequently continued to decrease over time. After 8 weeks, NRS scores had improved by ≥ 30% compared with baseline in 113 patients (69.3%). We divided the patients into two groups to identify factors associated with improvements in NRS scores of < 30% and ≥ 30% after 8 weeks (Table 4). There were no significant differences in age, sex, diagnosis, or BMI between groups, but the NeP score was significantly higher in patients who experienced pain relief < 30% than in those who experienced greater levels of pain relief (*p* = 0.0047). We examined whether there were differences in the adverse events and magnitude of NRS improvement between the patients with initial doses of 5 mg/day (*n* = 128) and 10 mg/day (*n* = 59). The incidence of adverse events in patients with an initial dose of 5 mg/day (50%, 64/128 patients) was not significantly different from that in patients with 10 mg/day (45.8%, 27/59 patients; *p* = 0.59). There were no significant differences in the magnitude of NRS improvement between the groups with initial doses of 5 mg/day (46.5% ± 24.8%) and 10 mg/day (45.5% ± 24.4%; *p* = 0.81). Similarly, there were no significant differences in the magnitude of NRS improvement in patients taking maximum doses of 5 mg/day (*n* = 47), 10 mg/day (*n* = 89), 20 mg/day (*n* = 32), and 30 mg/day (*n* = 19, *p* = 0.79; Fig. 2).

**Table 4.** Univariate analyses of patients with or without 30% pain relief by mirogabalin

**Variables**	**< 30% pain relief** **(*n*= 50)**	**≥ 30% pain relief** **(*n*= 113)**	***p*value**
Age (years)	72.9 ± 11.9 (45–88)	71.3 ± 13.0 (30–94)	0.46^a^
Sex, female/male	30/20	82/31	0.11^b^
Diagnosis			0.75^b^
LCS	35	83	
CSM	11	17	
LDH	2	7	
CTS	2	6	
Others	0	1	
BMI (kg/m^2^)	22.7 ± 4.2 (18–33)	23.0 ± 3.8 (19–31)	0.79^a^
DM	4	11	0.72^b^
NeP score (points)	7.4 ± 2.1 (6–12)	6.6 ± 1.4 (6–12)	0.0047^a^
Primary doze (mg)	3.1 ± 1.5 (5–10)	3.1 ± 1.1 (5–10)	0.97^a^
Max dose (mg)	6.6 ± 3.8 (5–30)	6.4 ± 3.8 (5–30)	0.76^a^
Adverse events	24 (48%)	42 (37.2%)	0.13^b^
Somnolence	17 (34%)	27 (23.9%)	
Dizziness	6 (12%)	10 (8.8%)	
Edema	2 (4%)	5 (4.4%)	
Weight gain	1 (2%)	0 (0%)	

Data are expressed as the mean ± standard deviation (range) or n (%).

*LCS* lumbar canal stenosis, *CSM* cervical spondylotic myelopathy, *LDH* lumbar disc herniation, *CTS* carpal tunnel syndrome, *BMI* body mass index, *DM* diabetes mellitus, *NeP* neuropathic pain

^a^ Student’s t-test

^b^ Chi-squared test

## Discussion

In this study, we used mirogabalin to treat patients with peripheral neuropathic pain who switched from pregabalin treatment to mirogabalin treatment due to lack of efficacy or adverse events. Mirogabalin is a new drug for the treatment of peripheral neuropathic pain, and to our knowledge, there have been no reports of its clinical outcomes for patients who were previously treated with pregabalin. Mirogabalin exerted a significant analgesic effect within 1 week and was associated with mild CNS adverse effects.

Guidelines for the pharmacologic management of neuropathic pain recommend drugs acting at α2δ subunits of voltage-gated calcium channels, serotonin–norepinephrine reuptake inhibitors, and tricyclic antidepressants as first-line agents, neurotropin and tramadol as second-line agents, and opioids as third-line agents [25]. Several other scientific associations and guidelines recommend gabapentinoids as first-line drugs for the treatment of neuropathic pain [26–29]. Mirogabalin, a ligand for the α2δ subunits (α2δ-1 and α2δ-2) of voltage-sensitive calcium channels in the CNS, was approved as a medication for pain relief in patients with peripheral neuropathic pain in 2019 in Japan. Mirogabalin reportedly relieved diabetic peripheral neuropathic pain in a dose-dependent manner in Asian patients with diabetic peripheral neuropathic pain and was associated with only mild adverse events [30]. It was also shown to be effective and well tolerated in the management of postherpetic neuralgia in Asian patients [31]. In the present study, which included only patients who withdrew from pregabalin treatment due to adverse events or lack of efficacy, we found that mirogabalin was an effective analgesic and that few patients stopped treatment due to adverse events.

In vitro studies of its pharmacologic action have demonstrated that mirogabalin had a higher binding affinity for the human and rat α2δ subunit than pregabalin [16]. In a dissociation rate analysis, the dissociation half-lives of mirogabalin from the α2δ-1 and α2δ-2 subunits were 11.1 h and 2.4 h, respectively, compared with 1.4 h for pregabalin at both subunits [16]. These reports indicated that mirogabalin has potent and selective binding affinities for the human and rat α2δ subunit and a slower dissociation rate for the α2δ-1 subunit than the α2δ-2 subunit compared with pregabalin [16]. These findings support our results that even patients who discontinued pregabalin because of its CNS effects experienced fewer adverse events when they were treated with mirogabalin. In this study, the initial dose was reduced to 5 mg/day in patients with impaired renal function or in the elderly patients. In patients with impaired renal function, we consider that the initial dose of 10 mg/day was too high, which may cause adverse events. However, in this study, the incidence of adverse events was nearly the same in the patients with impaired renal function as in the patients with normal renal function, and there was no significant difference in the magnitude of NRS improvement after 8 weeks of treatment. Therefore, we consider that administering a reduced dose is useful in patients with impaired renal function. It has also been reported that continued oral mirogabalin treatment increases the pain threshold over time [16]. In animal models of fibromyalgia, mirogabalin treatment has been shown to significantly decrease pain scores due to chronic allodynia [32]. Neuropathic pain results in higher pain scores than nociceptive pain [4]. In this study, the dose of mirogabalin was increased in response to pain over an 8-week treatment period. The magnitude of NRS improvement was 40% or more at the 10 mg/day, 20 mg/day, and 30 mg/day doses, and the analgesic effect of mirogabalin was evident after only 1 week, suggesting a good analgesic effect of mirogabalin.

The α2δ-1 subunit plays an important role in the onset and pathological persistence of neuropathic pain. Α2δ-1 expression levels correlated with tactile allodynia development were significantly increased in rats with spinal cord injury [33]. Knockdown of the α2δ-1 subunit by antisense oligodeoxynucleotides reportedly inhibited tactile allodynia in rat models [33, 34]. Overexpression of the α2δ-1 subunit resulted in enhanced currents, altered kinetics, and voltage-dependence of voltage-gated calcium channel activation in sensory neurons; exaggerated and prolonged dorsal horn neuronal responses to mechanical and thermal stimulations in the periphery; and enhanced pain-related behavior [35]. To the best of our knowledge, no study has addressed the association between the α2δ-2 subunit and pain. Edvardson et al. reported the importance of the α2δ-2 subunit, which is dominantly expressed in cerebellar Purkinje cells, in the normal physiology of the human brain [36]. Binding to the α2δ-1 subunit contributes to analgesic effects, whereas binding to the α2δ-2 subunit appears to contribute to undesirable CNS effects, such as somnolence [37–39]. These studies indicated that gabapentinoids exert their analgesic effects via the α2δ-1 subunit, and the α2δ-1 subunit thus plays a major role in neuropathic pain. These findings suggested that the α2δ-2 subunit may be implicated in the CNS adverse events commonly seen with pregabalin treatment. These findings also suggested that the selective actions of mirogabalin on the α2δ-1 and α2δ-2 subunits may maximize its analgesic effects while minimizing CNS adverse events. The potent binding affinity of mirogabalin with the α2δ-1 subunit and its long dissociation half-life from the α2δ-2 subunit may thus make mirogabalin an attractive agent for the treatment of peripheral neuropathic pain. Although 12.8% of patients in the present study discontinued treatment because of adverse events, mirogabalin was generally well tolerated.

Although the results of the present study suggest that mirogabalin might be an alternative treatment option for the treatment of peripheral neuropathic pain, the present study has some limitations. First, both pregabalin and mirogabalin are ligands for the α2δ subunit of voltage-sensitive calcium channels. However, this study had no pretrial protocol and is a case series without a comparison or placebo group. We did not compare these two drugs in this study. Thus, the results cannot be clearly attributed to mirogabalin administration. Second, this study had a short observation period. Although most patients treated with mirogabalin for 8 weeks maintained their weight within ± 5% of their baseline weight, weight gain can be an issue with mirogabalin when used for a longer period. A third limitation was a lack of determination of the best screening questionnaire for neuropathic pain. Neuropathic pain screening questionnaires include painDETECT [4], the spine painDETECT questionnaire, which is a screening tool for neuropathic pain caused by spinal disorders [40], and the NeP score used in this study. We considered that if the NeP scores were high, there would be little decrease in NRS scores after mirogabalin treatment, making it suitable as a baseline index. However, the use of other neuropathic pain screening questionnaires might have led to different results. We consider that the assessment and diagnosis of neuropathic pain should follow an identical algorithm that is widely used as a current international standard for the diagnosis of neuropathic pain, and it should specifically include (1) an assessment of the range of pain that is neuroanatomically plausible, (2) the suggestion of a lesion or disease of the somatosensory system, and (3) objective findings of sensory damage that are observed in the neuroanatomically innervated region of the damaged nerve or tests that are performed to provide a diagnosis of a neurological lesion or disease that accounts for the neuropathic pain. Despite these limitations, mirogabalin, a recently developed agent, showed promising results in patients with peripheral neuropathic pain.

## Conclusions

This investigation indicated that mirogabalin is safe and effective for reducing peripheral neuropathic pain in patients who ceased treatment with pregabalin due to adverse events or lack of efficacy.

## Data Availability

All data used and analyzed during this study are available from the corresponding author upon reasonable request.

## Abbreviations

QOL: Quality of life
CNS: Central nervous system
NRS: Numeric rating scale
BMI: Body mass index
